# Optimizing short-term in-class process evaluation: analyzing the effectiveness of teaching interventions in pharmaceutical education using repeated measures analysis

**DOI:** 10.1186/s12909-024-05754-y

**Published:** 2024-07-16

**Authors:** Leo Tsui, Yule Huang, Yihan Lei, Jin Wang

**Affiliations:** School of Pharmacy, Shanghai University of Medicine and Health Sciences, Shanghai, 201318 China

**Keywords:** Repeated measures analysis, Process evaluation, Pharmaceutical education, Pharmaceutical Care Network Europe, Pearson’s correlation

## Abstract

**Background:**

The assessment of the effectiveness of teaching interventions in enhancing students’ understanding of the Pharmaceutical Care Network Europe (PCNE) Classification System is crucial in pharmaceutical education. This is especially true in regions like China, where the integration of the PCNE system into undergraduate teaching is limited, despite its recognized benefits in addressing drug-related problems in clinical pharmacy practice. Therefore, this study aimed to evaluate the effectiveness of teaching interventions in improving students’ understanding of the PCNE Classification System in pharmaceutical education.

**Methods:**

Undergraduate pharmacy students participated in a series of sessions focused on the PCNE system, including lectures (t_1_), case analyses (t_2_), and practical implementation (t_3_). The levels of understanding were evaluated using time-course questionnaires. Initially, paired samples t-Tests were used to compare understanding levels between different time points. Subsequently, Repeated Measures Analysis (RMA) was employed. Pearson correlation analysis was conducted to examine the relationship between understanding levels and the usability and likelihood of using the PCNE system, as reported in the questionnaires.

**Results:**

The paired samples t-Tests indicated insignificant differences between t2 and t3, suggesting limited improvement following the practical implementation of the PCNE system. However, RMA revealed significant time effects on understanding levels in effective respondents and the focused subgroup without prior experience (random intercept models: all *p* < 0.001; random slope models: all *p* < 0.001). These results confirmed the effectiveness of all three teaching interventions. Pearson correlation analysis demonstrated significant positive correlations between understanding levels and the usability and likelihood of using the PCNE system at all examined time points. This finding highlighted the reliability of the understanding levels reported in the questionnaires. The homework scores were used as external calibration standards, providing robust external validation of the questionnaire’s validity.

**Conclusion:**

The implementation of RMA provided robust evidence of the positive impact of time on understanding levels. This affirmed the effectiveness of all teaching interventions in enhancing students’ comprehension of the PCNE Classification System. By utilizing RMA, potential errors inherent in common statistical methods, such as t-Tests, were mitigated. This ensured a more comprehensive and accurate assessment of the effectiveness of the teaching interventions.

**Supplementary Information:**

The online version contains supplementary material available at 10.1186/s12909-024-05754-y.

## Background

The assessment of student learning and comprehension is of utmost importance in pharmaceutical education, particularly when addressing complex topics such as the PCNE Classification System. This system is widely recognized for its clarity, validation methodology, and comprehensive outcomes, making it a valuable tool for addressing Drug-related Problems (DRPs) in clinical pharmacy practice [1]. The global adoption of the PCNE Classification System, supported by translations into various languages, including German and Bahasa Indonesia, highlights its usability and applicability in pharmaceutical care practice [2, 3]. Compared to other classification systems, the PCNE Classification System stands out due to its well-defined structure, rigorous validation process, and extensive use in clinical pharmacy practice worldwide [1]. However, despite its global acceptance, the integration of the PCNE Classification System into pharmaceutical education in China is still in its early stages, with limited emphasis on its application in undergraduate teaching.

Process evaluation plays a vital role in educational research by providing valuable insights into the implementation and effectiveness of teaching interventions [4]. It involves the systematic assessment of program delivery, fidelity, and contextual factors to understand the impact of educational strategies on student learning outcomes [5]. Process evaluation enables educators to identify strengths and weaknesses in instructional practices, guiding the refinement of teaching approaches to optimize learning outcomes. However, conducting process evaluation in pharmaceutical education presents unique challenges due to the complex nature of the subject matter and the need for timely feedback to inform instructional improvements.

Despite the global recognition and benefits of the PCNE Classification System in addressing drug-related problems, there has been limited integration of this system in undergraduate pharmaceutical education in China. This study aimed to address this gap by evaluating the effectiveness of teaching interventions designed to enhance students’ understanding of the PCNE system. In this study, RMA was employed for process evaluation to assess the implementation and effectiveness of teaching interventions within a condensed timeframe. RMA was used to analyze changes in outcomes over time within the same subjects, accounting for the correlation between repeated measurements [6]. By utilizing RMA alongside other statistical methods, changes in students’ understanding levels were assessed over time within a single class session. It was hypothesized that RMA would be a more appropriate statistical approach for process evaluation in teaching. This ensures a thorough comprehension of the influence of instructional practices on student learning outcomes.

## Methods

### Participants

The study involved undergraduate students majoring in pharmacy who were enrolled in the “Clinical Pharmacotherapy” course offered by two online classes at a university in Shanghai in 2022. This elective course is primarily intended for third-year students and spans 16 weeks, with 2 h of teaching per week. One of the sessions within the course focused on the PCNE classification system (Version 9.00) and lasted for two class hours. The session included a 20-minute lecture, a 20-minute case analysis, and a 40-minute practical implementation session, with 5-minute breaks in between (Supplemental Fig. 1). All students enrolled in the “Clinical Pharmacotherapy” course were invited to participate in the study. Those who did not consent to participate or were absent during the data collection sessions were excluded from the analysis. Participants were verbally informed that the questionnaire data would be anonymized and used solely for educational-related research purposes. They willingly completed time-course questionnaires at different intervals throughout the class, with the understanding that those who chose not to participate would be exempt from completing the questionnaires.

### Teaching interventions

The teaching intervention consisted of three main components: a lecture, case analysis, and practical implementation. Each component was designed to progressively enhance students’ understanding and application of the PCNE classification system. The lecture provided foundational knowledge about the PCNE classification system, including its purpose, structure, and key concepts. The objective of the lecture was to ensure that students could achieve a basic understanding of the system’s principles and terminology. By the end of the lecture, students were expected to have a foundational grasp of the system. Following the lecture, students participated in case analysis sessions. These sessions involved detailed discussions of clinical scenarios where students applied the PCNE classification system to identify and classify DRPs. The objective of the case analysis sessions was to deepen the students’ understanding and improve their ability to use the system in practical contexts. The case analysis aimed to develop students’ moderate understanding of the PCNE classification system’s application. In the final component, students engaged in hands-on practical exercises, implementing the system in simulated clinical settings. This stage sought to solidify their understanding and enhance their confidence in using the system independently. By the end of the practical implementation, students were expected to achieve high proficiency, finding the system simple to use and expressing a strong likelihood of utilizing it in future practice. The clear communication of objectives and expected learning outcomes at each stage ensured transparency and alignment with student learning goals. Throughout these stages, a progressive improvement in students’ understanding levels was anticipated, from foundational comprehension in the lecture to moderate understanding during case analysis, culminating in high proficiency by the end of practical implementation. In addition, to reinforce learning and practice, students were given a homework assignment following the course. This assignment required them to independently apply the PCNE classification system to additional case studies. The objective was to provide further practice with the system, ensuring a deeper understanding and retention of the material covered in the teaching interventions. This homework assignment served as a valuable exercise for consolidating their learning from the lecture, case analysis, and practical implementation sessions.

### Questionnaire design

Time-course questionnaires were structured to gauge understanding of the PCNE classification system at three distinct time points within the class: pre-class, in-class, and after-class (Supplemental Fig. 1). The pre-class questionnaire assessed participants’ gender, age, and prior experience with the PCNE classification system. The in-class questionnaire evaluated understanding levels of both the lecture (timepoint 1, t_1_) and case analysis (timepoint 2, t_2_). The after-class questionnaire focused on assessing understanding levels of practical implementation (timepoint 3, t_3_), as well as the usability of and likelihood of using the PCNE classification system in the future. Usability was defined as the ease with which students could use the PCNE classification system. High usability indicated that students could easily understand, operate, and apply what they learned without much extra effort. The likelihood of use was defined as the students’ willingness and possibility to apply the PCNE classification system they have learned in the future. A high “likelihood of use” indicated that students considered the PCNE classification system learned to be valuable to their future development and intended to apply it in real work. The response options for understanding levels included fully understanding, generally understanding, partially understanding, slightly understating, and beyond understanding. For usability, the response options included very easy to use, easy to use, average, not easy to use, and not at all easy to use. The response options for likelihood of using included definitely will, might, not sure, might not, and definitely will not. The questionnaires were conducted through an app and were independent of semester grades and graduation status. All students in the course used the app to receive and respond to questionnaires. Reminders were given only once, and participation was voluntary with no scoring. Each questionnaire allowed only one submission, and responses were unalterable after submission. Although no formal pilot study was conducted, the validity and reliability of the questionnaire were ensured through expert review, pretesting, design based on a validated instrument, test-retest reliability analysis through usability of and likelihood of use, and the use of homework scores as external calibration for validity. Additionally, to mitigate self-report and non-response bias, participants were informed consent that the analysis of the questionnaires was anonymized, voluntary participation was ensured, a well-established app was used, data were collected at multiple time points, clear instructions were provided, response rates were monitored, and statistical adjustments were applied.

### Data extraction and analysis

Questionnaire responses were extracted and reviewed by two independent reviewers. A fully answered questionnaire from the respondents was considered a valid response, and the focused group was defined as the effective respondents without pre-experience. Gender and pre-experience with the PCNE classification system were coded as binary data, while age was treated as numeric data. Participants could choose not to disclose personal information, but responses to the course understanding section were mandatory. Only one participant declined to disclose their age, and the missing data were replaced with the mean of overall participant ages. No other missing data were present. To enhance data interpretability, the ordinal data were recoded as follows: for understanding levels, fully understanding was recoded as 10, generally understanding as 7.5, partially understanding as 5, slightly understanding as 2.5, and beyond understanding as 0. Similarly, for usability, very easy to use was recoded as 10, easy to use as 7.5, average as 5, not easy to use as 2.5, and not at all easy to use as 0. For the likelihood of using the PCNE system, definitely will was recoded as 10, might as 7.5, not sure as 5, might not as 2.5, and definitely will not as 0. Data analysis, including counts, percentages, mean, and Standard Deviation (SD), was conducted using Microsoft 365 Excel.

### Paired samples t-test

The paired samples t-test was performed using the paired t.test() function in R software (version 4.2.3). The paired variables (e.g., t_2_ and t_1_, t_3_ and t_2_, t_3_ and t_1_) were specified as the two samples to be compared. The differences in changed understanding levels between two time-points (e.g., t_2_-t_1_ and t_3_-t_1_, t_3_-t_2_ and t_3_-t_1_, t_3_-t_2_ and t_2_-t_1_) were also calculated. A p-value < 0.05 was considered a statistically significant difference between two variables.

### Repeated measures analysis

RMA was conducted using R with the readxl and nlme packages. Understanding levels after the lecture, case analysis, and practical implementation were designated as t_1_, t_2_, and t_3_, respectively, for the same subjects. Random effects mixed models were employed to analyze understanding levels at different time points, with the time between t_1_, t_2_, and t_3_ considered an independent variable in the model. In the different models performed in this study, when the p-value of a variable in RMA is less than 0.05, it typically indicates that the variable resulted in a statistically significant difference in the understanding levels between different time-points. This suggests strong evidence that the observed differences in understanding levels across the various time-points are unlikely to have occurred due to random chance alone.

### Pearson’s correlation

Pearson’s correlation analysis was performed using the cor.test() function in R. The two variables, such as understanding levels at different time points (t_1_, t_2_, and t_3_) and changes in understanding levels (e.g., t_2_-t_1_ and t_3_-t_1_, t_3_-t_2_ and t_3_-t_1_, t_2_-t_1_ and t_3_-t_2_), were compared to usability or likelihood of using the PCNE system, respectively, as well as adjusted homework scores (basic points deducted from homework scores). The results were interpreted by examining Pearson’s Product-moment Correlation Coefficient (PPMCC), where values closer to 1 indicate a strong positive correlation between two variables, values closer to -1 indicate a strong negative correlation, and values close to 0 indicate little to no correlation. A p-value < 0.05 was considered a statistically significant correlation between two variables.

## Results

The pre-class questionnaire achieved an effective response rate of 94.7% (108 out of 114), while the in-class and after-class questionnaires had response rates of 89.6% (103 out of 115) and 93.0% (107 out of 115), respectively (Supplemental Fig. 1). After cross-analysis, 104 individuals completed the questionnaires, with 98 considered effective respondents (Table 1). The majority of respondents were female, accounting for 83.7% of total respondents and 87.8% of effective respondents. Male respondents constituted 16.3% and 12.2%, respectively. The highest proportion of participants were 21 years old, representing 56.7% of total respondents and 58.2% of effective respondents (Table 1). Participants aged 20 and 22 also made up significant portions, comprising 16.3% and 22.1% of total respondents, respectively (Table 1). Most respondents were undergraduates, constituting 86.5% of total respondents and 85.7% of effective respondents, while 13.5% had upgraded from junior college, with a similar percentage among effective respondents (Table 1). The majority of respondents (92.3% of total and 92.9% of effective) reported no prior experience with the PCNE classification system, while a small proportion (7.7% of total and 7.1% of effective) indicated having prior experience (Table 1).

**Table 1:** Demographic overview of participants responding to the questionnaires

Responded questionnaires	Respondents (*n* = 104)	Effective respondents (*n* = 98)
	*n* (%)	*n* (%)
**Gender**		
Female	87 (83.7)	86 (87.8)
Male	17 (16.3)	12 (12.2)
**Age**		
18	1 (1.0)	1 (1.0)
19	0 (0)	0 (0)
20	17 (16.3)	13 (13.3)
21	59 (56.7)	57 (58.2)
22	23 (22.1)	23 (23.5)
23	2 (1.9)	2 (2.0)
24	1 (1.0)	1 (1.0)
Refusal	1 (1.0)	1 (1.0)
**Education**		
Undergraduate	90 (86.5)	84 (85.7)
Upgraded from junior college	14 (13.5)	14 (14.3)
**Pre-experience**		
Yes	8 (7.7)	7 (7.1)
No	96 (92.3)	91 (92.9)

To assess teaching effectiveness during in-class evaluation, the understanding levels of effective respondents from time-course questionnaires were analyzed, alongside a focused subgroup lacking prior PCNE system experience (Table 2). Among the 98 effective respondents, understanding levels were assessed at three time points: t_1_ (after the lecture), t_2_ (after case analysis), and t_3_ (after practical implementation) (Table 2). Post-lecture (t_1_), most respondents reported being either generally understanding (39.8%) or partially understanding (50.0%) of the content (Table 2). Similar trends were observed post-case analysis (t_2_), with 58.2% indicating a generally understanding level and 36.7% reporting a partially understanding level (Table 2). Following practical implementation (t_3_), 57.1% stated a generally understanding level, while 34.7% reported a partially understanding level (Table 2). The focused subgroup analysis, comprising 91 respondents without pre-experience, yielded comparable results across all three time points, indicating consistent understanding levels (Table 2).

**Table 2:** Assessment of understanding levels in effective time-course questionnaires

Effective time-course questionnaires	Effective respondents (*n* = 98)	Focused group^¶^ (*n* = 91)
	*n* (%)	*n* (%)
**t**_**1**_: **after lecture**		
Fully understanding	3 (3.1)	2 (2.2)
Generally understanding	39 (39.8)	37 (40.7)
Partially understanding	49 (50.0)	46 (50.5)
Slightly understating	5 (5.1)	4 (4.4)
Beyond understanding	2 (2.0)	2 (2.2)
**t**_**2**_: **after case analysis**		
Fully understanding	3 (3.1)	2 (2.2)
Generally understanding	57 (58.2)	52 (57.1)
Partially understanding	36 (36.7)	35 (38.5)
Slightly understating	2 (2.0)	2 (2.2)
Beyond understanding	0 (0)	0 (0)
**t**_**3**_: **after practical implementation**		
Fully understanding	6 (6.1)	6 (6.6)
Generally understanding	56 (57.1)	50 (54.9)
Partially understanding	34 (34.7)	33 (36.3)
Slightly understating	2 (2.0)	2 (2.2)
Beyond understanding	0 (0)	0 (0)

^¶^the effective respondents without pre-experience

### Analysis of paired samples t-test

Among the 98 effective respondents and the 91 respondents in the focused subgroup, understanding levels were recalculated for quantitative analysis. The mean understanding levels at different time points (t_1_, t_2_, and t_3_) were provided alongside standard deviations (Table 3). Notably, the means of changed understanding levels were lower than the standard deviations for both effective respondents and the focus subgroup (Table 3), indicating notable variability in individual responses within the time-course questionnaires and underscoring the diversity of understanding levels among participants. Paired samples t-Tests evaluated the significance of differences between time points. For the overall effective respondents, significant differences were noted in understanding levels between t_1_ and both t_2_ (*p* < 0.0001) and t_3_ (*p* < 0.0001), but not between t_2_ and t_3_ (*p* = 0.469; Table 3). Similarly, within the focused subgroup without pre-experience, significant differences were found between t_1_ and both t_2_ (*p* < 0.0001) and t_3_ (*p* < 0.005), but not between t_2_ and t_3_ (*p* = 0.357; Table 3). Assessing the changes in understanding levels from t_1_ to t_3_ and from t_2_ to t_3_, statistically significant differences were detected in both instances for effective respondents and the focus subgroup (*p* < 0.0001; Table 3). However, while the difference from t_1_ to t_3_ and t_2_ to t_3_ was significant in effective respondents (*p* < 0.05), the difference was marginally insignificant in the focused subgroup (*p* = 0.0749; Table 3). These results indicate that understanding levels significantly improved from the initial assessment (t_1_) to subsequent time points (t_2_ and t_3_) for both effective respondents and the focused subgroup without pre-experience. However, the lack of statistically significant changes in understanding levels from t_2_ to t_3_ suggests that practical implementation of the PCNE system may not substantially impact students’ understanding levels, both among effective respondents and the focused subgroup.

**Table 3:** Analysis of quantified differences of understanding levels in effective time-course questionnaires

Timepoint	Effective respondents (*n* = 98)			Focused group^¶^ (*n* = 91)		
	Mean ± SD	Paired samples t-test		Mean ± SD	Paired samples t-test	
t_1_	5.92 ± 1.81	-	-	5.91 ± 1.77	-	-
t_2_	6.56 ± 1.46	*p* < 0.0001^‡^		6.48 ± 1.44	*p* < 0.0001^‡^	-
t_3_	6.68 ± 1.56	*p* < 0.0001^‡^	*p* = 0.469^#^	6.65 ± 1.59	*p* < 0.005^‡^	*p* = 0.357^#^
t_3_ - t_1_	0.77 ± 2.17	-	-	0.74 ± 2.09	-	-
t_2_ - t_1_	0.64 ± 1.31	*p* = 0.469^†^	-	0.58 ± 1.30	*p* = 0.357^†^	-
t_3_ - t_2_	0.13 ± 1.74	*p* < 0.0001^†^	*p* < 0.05^§^	0.16 ± 1.70	*p* < 0.0001^†^	*p* = 0.0749^§^

^‡^compared to t_1_; ^#^compared to t_2_; ^†^compared to t_3_ - t_1_; ^§^compared to t_2_ - t_1_; ^¶^the effective respondents without pre-experience; *SD* standard deviation

### Analysis of repeated measures analysis

To assess improvements in teaching effectiveness, particularly practical implementation (the change in understanding levels from t_2_ to t_3_), across different time points within a single class session, Repeated Measures ANOVA (RMA) was employed on understanding levels from the time-course questionnaires (Supplemental Fig. 2a and 2b). A random effects mixed model that integrated both random-intercept (Supplemental Fig. 3a and 3c) and random-slope effects (Supplemental Fig. 3b and 3d) of time was utilized to estimate the influence of time on understanding levels while considering fixed effects of time and other factors. The p-values derived from the fixed effects of factors in linear mixed-effects models are provided for different factors and their interactions. For effective respondents, the fixed effect of time significantly contributed to understanding levels, evident in both random-intercept (*p* < 0.001) and random-slope effects (*p* < 0.001) in random effects mixed models (Table 4). Additionally, for the single factor of gender, the fixed effect significantly influenced understanding levels with random-slope effects (*p* < 0.05) in a random effects mixed model (Table 4). Similarly, within the focus subgroup, both the random-intercept and random-slope effects models yielded p-values less than 0.001 in the single factor analysis of time, indicating significant changes in understanding levels over time. Moreover, for the single factor of gender, both models resulted in p-values less than 0.05, suggesting a notable influence of gender on understanding levels, particularly within the focus group (Table 4). No other single, bi-, or multiple factors significantly impacted understanding levels in random effects mixed models using RMA (Table 4). Overall, the results from the RMA affirm the validity of the effect of time on understanding levels derived from the time-course questionnaires.

**Table 4:** Repeated-measures analysis using random effects mixed models for the respondents without pre-experience in effective time-course questionnaires

The *p*-value derived from fixed effects of the factor in a linear mixed-effects model	Effective respondents (*n* = 98)		Focused group^¶^ (*n* = 91)	
	Radom-intercept effects model	Random-slope effects model	Radom-intercept effects model	Random-slope effects model
**Single factor: time**				
(Intercept)	*p* < 0.001	*p* < 0.001	*p* < 0.001	*p* < 0.001
Time	*p* < 0.001	*p* < 0.001	*p* < 0.001	*p* < 0.001
**Single factor: gender**				
(Intercept)	*p* < 0.001	*p* < 0.001	*p* < 0.001	*p* < 0.001
Gender	*p* = 0.102	*p* < 0.05	*p* < 0.05	*p* < 0.05
**Single factor: age**				
(Intercept)	*p* = 0.171	*p* = 0.157	*p* = 0.322	*p* = 0.286
Age	*p* = 0.625	*p* = 0.597	*p* = 0.417	*p* = 0.429
**Single factor: pre-experience**				
(Intercept)	*p* < 0.001	*p* < 0.001	-	-
Pre-experience	*p* = 0.256	*p* = 0.206	-	-
**Bi-factor: time and gender**				
(Intercept)	*p* < 0.001	*p* < 0.001	*p* < 0.001	*p* < 0.001
Time	*p* < 0.001	*p* < 0.001	*p* < 0.001	*p* < 0.001
Gender	*p* = 0.717	*p* = 0.776	*p* = 0.577	*p* = 0.655
Interaction: time and gender	*p* = 0.112	*p* = 0.191	*p* = 0.342	*p* = 0.433
**Bi-factor: time and age**				
(Intercept)	*p* = 0.443	*p* = 0.545	*p* = 0.778	*p* = 0.819
Time	*p* = 0.978	*p* = 0.982	*p* = 0.710	*p* = 0.759
Age	*p* = 0.861	*p* = 0.900	*p* = 0.513	*p* = 0.597
Interaction: time and age	*p* = 0.897	*p* = 0.916	*p* = 0.827	*p* = 0.857
**Bi-factor: time and pre-experience**				
(Intercept)	*p* < 0.001	*p* < 0.001	-	-
Time	*p* < 0.001	*p* < 0.005	-	-
Pre-experience	*p* = 0.789	*p* = 0.833	-	-
Interaction: time and pre-experience	*p* = 0.638	*p* = 0.700	-	-
**Multi-factor: time**,** gender and pre-experience**				
(Intercept)	*p* < 0.001	*p* < 0.001	-	-
Time	*p* < 0.001	*p* < 0.001	-	-
Gender	*p* = 0.742	*p* = 0.798	-	-
Pre-experience	*p* = 0.825	*p* = 0.863	-	-
Interaction: time and gender	*p* = 0.095	*p* = 0.171	-	-
Interaction: time and pre-experience	*p* = 0.485	*p* = 0.567	-	-
**Multi-factor: time**,** gender**,** age and pre-experience**				
(Intercept)	*p* = 0.443	*p* = 0.551	-	-
Time	*p* = 0.937	*p* = 0.949	-	-
Gender	*p* = 0.751	*p* = 0.805	-	-
Age	*p* = 0.864	*p* = 0.894	-	-
Pre-experience	*p* = 0.818	*p* = 0.858	-	-
Interaction: time and gender	*p* = 0.094	*p* = 0.170	-	-
Interaction: time and age	*p* = 0.801	*p* = 0.837	-	-
Interaction: time and pre-experience	*p* = 0.477	*p* = 0.561	-	-

^¶^the effective respondents without pre-experience

### Analysis of pearson’s correlation

To assess the internal consistency and ensure the reliability of the understanding levels reported in the time-course questionnaires, the usability and likelihood of using the PCNE classification system were evaluated among both effective respondents and the focused group. Results indicated that a majority of participants found the PCNE system easy to use after practical exercises, with 16.3% and 15.4% of effective respondents and the focused group, respectively, rating it as very easy, and 58.2% and 57.1% as easy to use (Table 5). Similarly, most respondents expressed a likelihood of using the PCNE system in the future, with 68.4% of effective respondents and 70.3% of the focused group indicating they might use it (Table 5). Only a small percentage of respondents were uncertain about future use (Table 5).

**Table 5:** Usability of and likelihood of using the PCNE classification system from the effective respondents and the focused group

Effective time-course questionnaires	Effective respondents (*n* = 98)	Focused group^¶^ (*n* = 91)
	*n* (%)	*n* (%)
**Usability of the PCNE classification system after practical exercises**		
Very easy to use	16 (16.3)	14 (15.4)
Easy to use	57 (58.2)	52 (57.1)
Average	25 (25.5)	25 (27.5)
Not easy to use	0 (0)	0 (0)
Not at all easy to use	0 (0)	0 (0)
**Likelihood of using the PCNE classification system in the future**		
Definitely will	21 (21.4)	17 (18.7)
Might	67 (68.4)	64 (70.3)
Not sure	10 (10.2)	10 (11.0)
Might not	0 (0)	0 (0)
Definitely will not	0 (0)	0 (0)

*PCNE Pharmaceutical Care Network Europe, ^¶^the effective respondents without pre-experience

Pearson’s correlation coefficient was used to examine the relationship between understanding levels at different time-points (t_1_, t_2_, and t_3_) and the usability and likelihood of using the PCNE system. Significant positive correlations were found between understanding levels and both usability and likelihood of future use for both effective respondents and the focused group (all *p* < 0.05; Table 6). For both groups, there were no significant correlations between changes in understanding levels at different time points and the usability or likelihood of using the PCNE system. The only exception was in the focused group, where a significant correlation was found between the change in understanding from t_2_ to t_3_ and perceived usability of the PCNE system (*p* < 0.05; Table 6). These findings demonstrate a positive association between understanding levels and the usability and likelihood of using the PCNE classification system. Moreover, they indicate that differences in understanding levels are not significantly linked to the usability or likelihood of using the PCNE system. Overall, these results reinforce the reliability of the understanding levels reported in the time-course questionnaires and emphasize the importance of employing RMA to comprehensively evaluate understanding levels following various teaching activities over time.

**Table 6:** Correlation analysis of changed understanding level compared to usability of and likelihood of using the PCNE classification system from the effective respondents and the focused group

Pearson’s correlation	Effective respondents (*n* = 98)		Focused group^¶^ (*n* = 91)	
	PPMCC	Correlation test	PPMCC	Correlation test
t_1_ - usability	0.361	*p* < 0.001	0.340	*p* < 0.001
t_1_ - likelihood	0.205	*p* < 0.05	0.187	*p* = 0.076
t_2_ - usability	0.401	*p* < 0.001	0.373	*p* < 0.001
t_2_ - likelihood	0.260	*p* < 0.01	0.243	*p* < 0.05
t_3_ - usability	0.594	*p* < 0.001	0.628	*p* < 0.001
t_3_ - likelihood	0.317	*p* < 0.005	0.334	*p* < 0.005
t_2_-t_1_ - usability	-0.052	*p* = 0.610	-0.048	*p* = 0.648
t_2_-t_1_ - likelihood	0.007	*p* = 0.946	0.015	*p* = 0.886
t_3_-t_2_ - usability	0.195	*p* = 0.054	0.271	*p* < 0.01
t_3_-t_2_ - likelihood	0.065	*p* = 0.522	0.107	*p* = 0.314
t_3_-t_1_ - usability	0.125	*p* = 0.220	0.190	*p* = 0.071
t_3_-t_1_ - likelihood	0.057	*p* = 0.580	0.096	*p* = 0.365

*PCNE Pharmaceutical Care Network Europe, *PPMCC* Pearson’s product moment correlation coefficient, ^¶^the effective respondents without pre-experience

To assess the questionnaire’s validity, homework scores were used as external calibration standards, which serve as an objective and quantifiable measure reflecting students’ actual performance on understanding levels. By correlating the questionnaire’s understanding levels with students’ homework scores, the aim was to validate whether self-assessment effectively captured students’ learning outcomes. Following the completion of the questionnaires, data on students’ homework scores was collected, and Pearson’s correlation analysis was conducted to examine the relationship between questionnaire-derived understanding levels and actual homework performance. Significant positive correlations were observed between the changes in understanding levels reported in the questionnaires and students’ homework scores for both the effective respondents and the focused group (Table 7). This correlation analysis substantiated that the changes in understanding levels assessed through the questionnaire corresponded with students’ performance as reflected in their homework scores (Table 7). Overall, using homework scores as external calibration standards provided robust external validation of the questionnaire’s validity, thereby ensuring the accuracy and reliability of the measurement results.

**Table 7:** Correlation analysis of changed understanding level compared to homework score from the effective respondents and the focused group

Pearson’s correlation	Effective respondents (*n* = 98)		Focused group^¶^ (*n* = 91)	
	PPMCC	Correlation test	PPMCC	Correlation Test
t_1_ - homework score	-0.155	*p* = 0.129	-0.149	*p* = 0.159
t_2_ - homework score	-0.141	*p* = 0.166	-0.112	*p* = 0.290
t_3_ - homework score	0.097	*p* = 0.344	0.113	*p* = 0.286
t_2_-t_1_ - homework score	0.056	*p* = 0.582	0.079	*p* = 0.459
t_3_-t_2_ - homework score	0.205	*p* < 0.050	0.201	*p* = 0.056
t_3_-t_1_ - homework score	0.198	*p* = 0.050	0.212	*p* < 0.050

*PPMCC Pearson’s product moment correlation coefficient, ^¶^the effective respondents without pre-experience

## Discussion

In this study, which was conducted in a single class session, the application of RMA proved invaluable for comprehensively assessing the effectiveness of teaching activities. Unlike traditional t-tests, RMA enabled a nuanced examination of changes in understanding levels over time, thereby enhancing the reliability and validity of the findings. This approach facilitated timely adjustments to teaching strategies based on real-time insights, maximizing instructional efficacy. The adoption of RMA in short-term teaching contexts held significant practical implications and offered generalizable value, ensuring more robust evaluations of instructional interventions and effectively informing pedagogical practices. By utilizing RMA, educators could gain deeper insights into students’ comprehension levels across various teaching activities over time, particularly for process evaluation purposes in short-term teaching contexts.

The study emphasizes the reliability of understanding levels assessed through time-course questionnaires, as evidenced by their positive correlations with the PCNE classification system’s usability and future adoption likelihood. The majority of participants expressed positive attitudes toward the PCNE system and indicated plans to use it in the future. Across all examined time points, consistently significant positive correlations were observed between understanding levels, the system’s usability, and future adoption likelihood. Variations in understanding levels over time, however, did not consistently align with the system’s usability or adoption likelihood, except for specific instances in the focused group. This suggests that participants predominantly base their assessments of system usability and future adoption willingness on personal feelings rather than societal expectations, highlighting the importance of understanding individual intrinsic feelings and experiences in research.

Additionally, the significant variability in individual responses within the time-course questionnaires, with the means of changed understanding levels being notably lower than the standard deviations for both effective respondents and the focus subgroup, underscores the intricate nature of the learning process. Upon reviewing the raw data, it became evident that many participants did not consistently report an increase in their understanding levels over time based on social expectations; instead, some experienced negative growth in their reported understanding levels. This emphasizes the crucial need for tailored and effective instructional strategies to address diverse learner needs, highlighting the importance of meticulous interpretation and the application of RMA in the process evaluation of learning outcomes.

RMA provided a robust methodological approach to capture changes in students’ comprehension levels over multiple time points within a single class session. This approach enabled a comprehensive evaluation of students’ understanding levels following different teaching activities related to the PCNE Classification System, such as lectures, case analyses, and practical implementations. By analyzing the variation in understanding levels over time, the effectiveness of these teaching interventions in enhancing students’ comprehension within a condensed timeframe could be discerned. The incorporation of RMA into the assessment framework was particularly relevant given the limited time dedicated to teaching the PCNE Classification System. Despite the course in clinical pharmacotherapy spanning 16 weeks, with the PCNE Classification System being covered in only one week, traditional assessment methods might fail to capture the nuanced changes in students’ comprehension levels throughout the class session. In contrast, RMA allowed for a more nuanced examination of students’ understanding, facilitating the identification of areas for improvement and the refinement of teaching strategies within the limited timeframe. Moreover, the application of RMA facilitated the identification of trends and patterns in students’ learning trajectories over time. This longitudinal perspective provided valuable insights into the dynamics of knowledge acquisition and retention within a single-class session. By analyzing the temporal progression of students’ comprehension levels, educators could gain a deeper understanding of the effectiveness of teaching interventions and tailor instructional approaches to better meet students’ learning needs.

While traditional testing methods are valuable for evaluating students’ learning achievements [7], they may not effectively assess mastery of clinical application tools, such as the PCNE classification system. For instance, Strandell-Laine et al. developed a mobile application to facilitate collaboration between nursing students and nurse teachers during clinical practicum, aiming to enhance students’ clinical learning outcomes [8]. Their process evaluation, conducted through questionnaires and essays completed by second-year nursing students, focused on assessing the usability and acceptability of the mobile application [8]. Compared to their study, our research provides a more detailed analysis of teaching effectiveness within a limited timeframe. Using RMA, we were able to capture subtle variations in students’ comprehension levels throughout the instructional period. This approach offers valuable insights into the immediate impact of teaching interventions and enhances our understanding of instructional practices in pharmaceutical education, which allows for targeted interventions to optimize student learning outcomes. Moreover, Unger et al. explored the use of self-recorded video portfolios to help physiotherapy students achieve competence in performing techniques, evaluated through self-perception questionnaires and RMA [9]. The results of their study align with our findings, highlighting the effectiveness and reliability of using self-assessment tools and RMA in educational intervention evaluations [9]. Although our study focused on the PCNE classification system for short-term in-class process evaluation, while theirs focused on physiotherapy techniques for long-term evaluation, both studies demonstrate the potential of RMA in assessing educational interventions.

For future research, a mixed-methods approach that integrates subjective questionnaire data with objective measurements or qualitative data is recommended. This approach will allow researchers to triangulate findings, validate results, and capture a more comprehensive understanding of students’ comprehension levels as well as the impact of teaching interventions. Additionally, longitudinal studies that follow students over an extended period can be conducted to assess the long-term retention of knowledge and skills acquired through educational interventions, providing insights into the sustainability of learning outcomes over time. By employing rigorous research methodologies and considering the limitations of each approach, researchers can contribute to advancing the understanding of teaching effectiveness and student learning in pharmaceutical education.

### Limitations

Several limitations of the study are worth noting. The sequential and segmented nature of the learning activities presented challenges when a repeated-measures design is employed for educational evaluation [10–12]. The condensed timeframe limited the depth of exploration for each topic, potentially impacting the thoroughness of students’ comprehension. External factors, such as individual learning styles and prior knowledge, may have influenced the results. Additionally, the study was confined to undergraduate pharmacy students from a specific university in Shanghai, China. This could limit the generalizability of the findings to other educational settings or student cohorts. Variations in curriculum structure, teaching methodologies, and student demographics across institutions may affect the applicability of the results.

Furthermore, while the RMA offered valuable insights into students’ understanding levels over time, the statistical methodology employed in this study may have limitations, including assumptions underlying the RMA. It is recognized that various learning activities contribute to enhancing understanding levels [13–15]. Addressing these limitations in future research endeavors will improve our understanding of teaching interventions that incorporate RMA in pharmaceutical education.

## Conclusion

RMA emerged as a robust method for evaluating instructional interventions. The study underscored the significance of RMA in short-term teaching contexts, particularly in focused sessions like the one covering the PCNE Classification System within a single class session. By tracking changes in students’ understanding levels throughout the session, the impact of teaching activities on learning outcomes was comprehensively gauged. This approach not only enhanced the understanding of teaching effectiveness but also allowed for timely adjustments to meet students’ learning needs. The insights gleaned from this analysis contributed to assessing teaching effectiveness and designing interventions to improve student learning outcomes within limited instructional time-frames.

### Electronic supplementary material

Below is the link to the electronic supplementary material.

Supplementary Material 1

## Data Availability

All data from the questionnaires in this study have been delinked, and the delinked data are provided in the Supplementary Materials.

## Abbreviations

DRP: Drug-related problems
PCNE: Pharmaceutical Care Network Europe
PPMCC: Pearson’s product moment correlation coefficient
RMA: Repeated measures analysis
