# Supplementary material for: Optimizing short-term in-class process evaluation: analyzing the effectiveness of teaching interventions in pharmaceutical education using repeated measures analysis
Source: BMC Med Educ. 2024 Jul 16;24:765. doi: 10.1186/s12909-024-05754-y (PMC11253344; doi:10.1186/s12909-024-05754-y)

Supplemental Figure 1 Design of repeated-measures in time-course questionnaires.

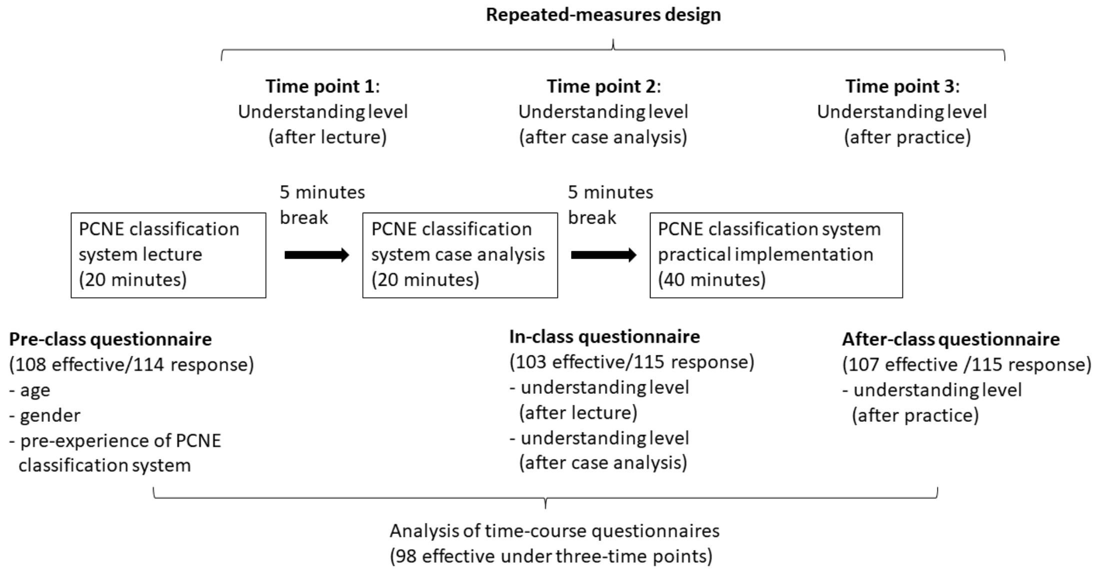

Supplemental Figure 2 The re-coded data from the time-course questionnaires for repeated-measures analysis. (a) Overall effective respondents (n = 98). (b) The focus group (n = 91).

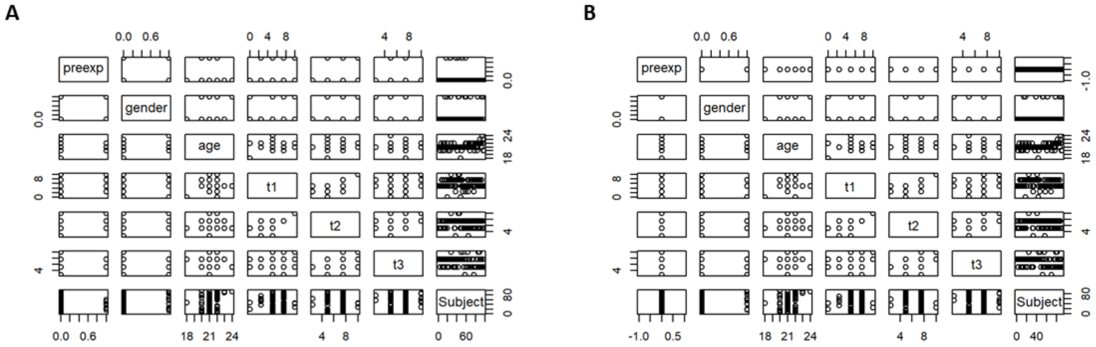

Supplemental Figure 3 Random effects mixed model with single factor analysis of time. (a) random-intercept effects model of effective respondents. (b) random-slope effects model of effective respondents. (c) random-intercept effects model of the focus group. (d) random-slope effects model of the focus group.

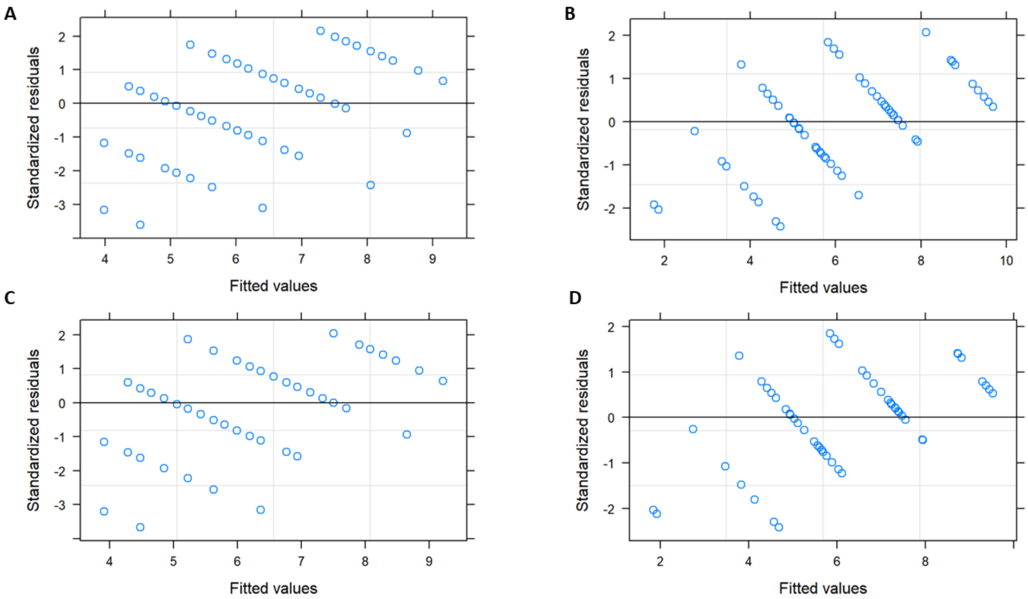

Supplement: Supplementary file 1 — Supplementary Material 1 [file 12909_2024_5754_MOESM1_ESM.pdf]
